# The Impact of Human Activities on Microbial Quality of Rivers in the Vhembe District, South Africa

**DOI:** 10.3390/ijerph13080817

**Published:** 2016-08-12

**Authors:** Afsatou N. Traoré, Khodani Mulaudzi, Gamuchirai J.E. Chari, Stefan H. Foord, Lutendo S. Mudau, Tobias G. Barnard, Natasha Potgieter

**Affiliations:** 1 Microbiology Department, University of Venda, Private Bag X5050, Thohoyandou 0950, South Africa; khodanim43@gmail.com (K.M.); gjechari@yahoo.com (G.J.E.C.); natasha.potgieter@univen.ac.za (N.P.); 2 Zoology Department, University of Venda, Private Bag X5050, Thohoyandou 0950, South Africa; Stefan.Foord@univen.ac.za; 3 Department of Environmental Health, Tshwane University of Technology, Private Bag X680, Pretoria 0001, South Africa; MudauLS@tut.ac.za; 4 Water & Health Research Unit, University of Johannesburg, PO Box 524, Auckland Park, Johannesburg 2006, South Africa; tgbarnard@uj.ac.za; 5 Dean, School of Mathematical and Natural Sciences, University of Venda, Private Bag X5050, Thohoyandou 0950, South Africa

**Keywords:** quanti-tray, total coliforms, *E. coli*, PCR, risk assessment

## Abstract

**Background:** Water quality testing is dictated by microbial agents found at the time of sampling in reference to their acceptable risk levels. Human activities might contaminate valuable water resources and add to the microbial load present in water bodies. Therefore, the effects of human activities on the microbial quality of rivers collected from twelve catchments in the Vhembe District in South Africa were investigated, with samples analyzed for total coliform (TC) and *Eschericha coli* (*E. coli)* contents. **Methods:** Physical parameters and various human activities were recorded for each sampling site. The Quanti-Tray^®^ method was adopted for the assessment of TC and *E. coli* contents in the rivers over a two-year period. A multiplex polymerase chain (PCR) method was used to characterize the strains of *E. coli* found. **Results:** The microbial quality of the rivers was poor with both TC and *E. coli* contents found to be over acceptable limits set by the South African Department of Water and Sanitation (DWS). No significant difference (*p* > 0.05) was detected between TC and *E. coli* risks in dry and wet seasons. All six pathogenic *E. coli* strains were identified and Enteroaggregative *E. coli* (EAEC), atypical Enteropathogenic *E. coli* (a-EPEC) and Enterotoxigenic *E. coli* (ETEC) were the most prevalent *E. coli* strains detected (respectively, 87%, 86% and 83%). **Conclusions:** The study indicated that contamination in the majority of sampling sites, due to human activities such as car wash, animal grazing and farming, poses health risks to communities using the rivers for various domestic chores. It is therefore recommended that more education by the respective departments is done to avert pollution of rivers and prevent health risks to the communities in the Vhembe District.

## 1. Introduction

The Vhembe district, situated in the Limpopo province of South Africa, is a poverty-stricken province, mainly a rural community in which the majority of people are subsistence farmers. Human and animal activities in this area contribute greatly to the contamination of the various rivers [1,2,3]. In addition, people in the district rely heavily on these rivers as sources for drinking and irrigation purposes.

However, waters from these rivers are highly contaminated with pathogenic organisms varying from microorganisms to parasites [4]. Many of these are pathogenic and lead to detriment of human health [4]. Studies have shown that human and animal activities could impact the quality of water sources [5,6]. The major source of microbial contamination arises from poor sanitation in developing areas. Most of these areas lack adequate excreta disposal [7]. Developing areas are well known for the lack of hygiene which only serves to compound the problem of poor water quality especially in rural areas [3]. This leads to an increasing risk of contracting bacterial diarrheal diseases. Other human activities that contribute to contamination includes bathing and washing of clothes in river sources. Of particular concern, is the presence of pathogenic bacteria that are the leading cause of gastrointestinal diseases in humans. *Eschericia coli (E. coli)* is a bacterial species that is widely used and accepted as an indicator organism in the testing of water quality all over the world. Its presence has proven to be the best indicator for fecal contamination of water supplies especially in open sources such as rivers [8]. It is also a leading contributor to enteric and diarrheagenic diseases especially in young children in developing countries [9].

The standard of water quality is often dictated by the number of microbial agents found in accordance with acceptable risk levels determined by legislature and WHO guidelines [10]. However, the absence of indicator organisms in water does not guarantee safety of drinking water [11]. The presence of bacteria and pathogenic organisms is of concern when considering the safety and quality of water [12]. Although *E. coli* might be detected in the water samples indicating the possible presence of other fecal related bacterial pathogens, it must be kept in mind that *E. coli* itself has seven distinct pathogenic groups capable of causing diarrhea [13,14]. The five *E. coli* pathotypes chosen by Omar and Barnard to target related virulence genes, using multiplex PCR include Enteropathogenic *E. coli* (EPEC), Enterotoxigenic *E. coli* (ETEC), Enterohaemorrhagic *E. coli* (EHEC), Enteroaggregative *E. coli* (EAEC) and Entero-invasive *E. coli* (EIEC) [15]. The addition of the mPCR to the Colilert^®^ (IDEXX, Westbrook, ME, USA) method offers a glimpse into the *E. coli* community present in a specific sample and also points out the possible increased risk of infection.

The present study aims to evaluate the impact of human activities on the quality of selected rivers and their catchments in the Vhembe District and to evaluate health risks associated with the consumption of such contaminated waters.

## 2. Experimental Section

### 2.1. Study Area and Sample Collection

**Figure 1:**
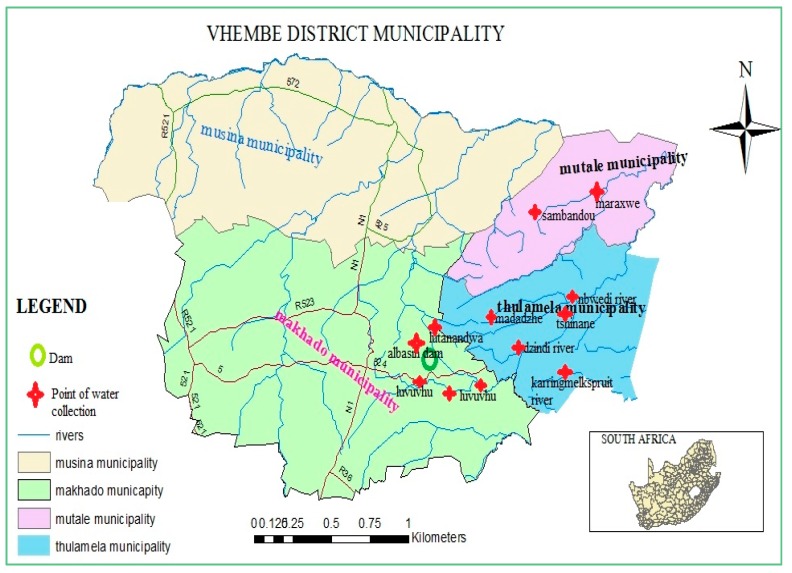
The Vhembe district municipality and sampling points in the Limpopo province, South Africa.

The study was conducted in the Vhembe district of Limpopo, South Africa. It involved 6 river sources from different parts of this district. The rivers included Mutale (Mut), Sambandou (Sam), Mbwedi (Mbw), Tshinane (Tsh), Madadzhe (Mad) and various catchments of the Luvuvhu river such as Tshino (Tshi), Dzindi (Dz), Lutanandwa (Lu), Karringmelkspruit (Kar), Springflied (Spr), Piesangoek (Pie) and the Albasin Dam (Albas) (Figure 1).

A total of 96 water samples (12 sampling points × upstream (Up) and downstream (Dw) × 2 sample collection trips each year (dry and wet seasons) were taken over a 2-year period from the selected sites in sterile 500 mL sampling bottles and stored at 4 °C on route to the laboratory. Samples were collected in the dry season (before the rains, July and September) and again in the rainy season (October and November). Samplings were done at two points (upstream and downstream as points located 100 m, respectively, before and after specific human activities) in each river site. The anthropogenic land uses at each point were observed and noted (Table 1). Temperature (°C), electrical conductivity (EC) (μS/cm), Total Dissolved Solids (TDS) (mg/L) and pH were measured in situ using a multimeter Crison Multimeter MM40 (Crison, Spain).

### 2.2. Detecting the Presence of Indicator Organisms in River Samples

The Colilert Quanti-Tray/2000 (IDEXX) system was used to estimate the most probable number of *E. coli* per 100 mL of river waters as per manufacturer’s instructions. The Quanti-Trays were incubated at 35 °C for 18 h. All positive wells for total coliforms were yellow and recorded for each sample. The Quanti-Trays were then examined under long wave (366 nm) ultraviolet light, and fluorescent wells were recorded as *E. coli* positive. Controls included the Quanti-Cult reference strains (*E. coli*, *Klebsiella pneumoniae* and *Pseudomonas aeruginosa*) as well as a water blank run concurrently with the samples. No dilutions of the samples were done and the results are reported as most probable number (MPN) for 100 mL sample analysed.

Ten (10) positive *E. coli* wells of each Colilert Quanti-Trays were randomly selected and a total of 2 mL of media was removed using a 1 mL disposable syringe and aliquoted into sterile Eppendorff tubes and stored at −20 °C until further analysis.

### 2.3. Identification of Pathogenic E. coli Strains

#### 2.3.1. Growth and Maintenance of Bacterial Strains

All bacterial strains (Table 2) used for the microbiology and PCR experiments were cultured on Plate Count Agar (PCA) from Oxoid, UK and incubated under aerobic conditions at 37 °C for 16 h. Single colonies were enriched in nutrient broth (Oxoid, UK) and incubated in a shaking incubator (150 rpm) under aerobic conditions at 37 °C for 16 h.

#### 2.3.2. Characterization of *E. coli* Communities with Multiplex PCR (m-PCR)

This study used the m-PCR method published by Omar and Barnard for the confirmation and characterization of the *E. coli* communities in the *E. coli* positive samples [15]. The m-PCR included virulence genes of the six *E. coli* groups, namely commensal *Escherichia coli* (CEC), Enteropathogenic *E. coli* (EPEC), Enterohaemorrhagic *E. coli* (EHEC), Enterotoxigenic *E. coli* (ETEC), Enteroinvasive *E. coli* (EIEC) and Enteroaggregative *E. coli* (EAEC). The specific genes targeted are the *mdh*-(*E. coli* housekeeping gene), *ial*-(EIEC), *eaeA* (EHEC and atypical EPEC), *bfp*-(typical EPEC), *eagg*-(EAEC), *stx1*- and *stx2*-(EHEC), *lt*- and *st*-(ETEC), *asta*-(*E. coli* toxin) and *gapdh* gene (external control). The primers used in the m-PCR for the amplification of these genes were procured from Inqaba Biotechnologies (Pty) Ltd. (Johannesburg, South Africa) and Whitehead Scientific (Pty) Ltd. (Johannesburg, South Africa).

All PCRs were performed in a BIO-RAD^®^ T100^TM^ thermal Mycycler following the 11-gene multiplex PCR (m-PCR) protocol optimised by Omar et al. [16]. The samples were amplified in a 20 µL reaction mixture containing 10 µL of the 2 × Qiagen^®^ m-PCR master mix (Hotstart Taq DNA polymerase, 10 × buffer, 2 mM MgCl_2_ and dNTP mix), 1 µL 5 × Q-solution, 4.5 µL of PCR grade water, 2 µL MgCl_2_, 2 µL of template DNA and 0.5 µL of the primer mix (0.1 µM of *mdh* and *lt* primers (F and R), 0.2 µM of *ial*, *gapdh*, *eagg, asta*, and *bfp* primers (F and R), 0.3 µM of *eaeA* and *stx2* primers (F and R), 0.5 µM of *stx1* and *st* primers (F and R)).

All PCR reactions were performed using enzyme activation at 95 °C for 15 min, 35 cycles of DNA denaturation at 94 °C for 45 s, annealing at 55 °C for 45 s, elongation at 68 °C for 2 min with a final elongation step at 72 °C for 5 min. For the negative control reaction mixture, the template DNA was replaced with sterile PCR grade water and the positive reaction contained DNA from *E. coli* reference strains (Table 2).

#### 2.3.3. Visualization of PCR Products

All DNA samples were separated in a horizontal agarose gel slab (2.5% (*w*/*v*)) containing ethidium bromide (0.5 µg/mL) using TAE buffer (40 mM Tris acetate, 2 mM EDTA, pH 8.3). Electrophoresis was conducted at 80–100 Volts for 1–2 h and viewed under UV light (Gene Genius Bio Imaging System, Vacutec^®^, Costa Mesa. CA, USA). The relevant sizes of the DNA fragments were estimated by comparing their electrophoretic mobility to that of a standard 100 bp and positive control that was run with the samples on each gel. This procedure was carried out for all experiments.

### 2.4. Risk Assessment Analysis

The analysis was determined taking into consideration upstream and downstream measurements taken during the dry and wet seasons. The observation was used to determine risk activities likely to contaminate water on-site or nearby catchments. To determine the decrease or increase of physical parameters, correlation measurements were carried out. The results for the physico parameters were assessed using acceptable standards set by the South African Water Standard (SANS) 241-2011, for pH (6–9), EC (70–350 μS/cm) and TDS (1000 mg/L) [17].

Total coliform (TC) and *E. coli* (EC) contents were analyzed on water samples and risk assessment scores determined according to the World Health Organization: TC and *E. coli* contents have low risk contamination (1–3) when equal 1 to 10 MPN/100 mL; intermediate to high risk (4–6) equates to 11 to 100 MPN/100 mL; and when MPN/100 mL is >100, the contamination risk is very high (7–10) [18].

### 2.5. Data and Statistical Analyses

Total Coliform (TC) and *E. coli* variations were firstly normalized and then modeled with linear mixed models using the “lmer” function in the lme4 package in R [19,20]. Sites were included as random factors to account for temporal pseudo replication. Fixed factors included TDS, EC, pH and Month, while Farming, Human activities (bathing and washing), and the presence of cattle were included as dummy variables. Model residuals were inspected for normality, constant variance and independence. The performance of combinations of explanatory variables was explored using the function “dredge” in the package “MuMln” using the bias corrected Akaike information criterion (AICc), which was then used to select the best model [21,22].

The *t*-test was used to assess for significant differences in the prevalence of TC and *E. coli* contents between dry and wet seasons and *p* < 0.05 was seen as significant.

## 3. Results and Discussion

### 3.1. Physical Parameters

**Figure 2:**
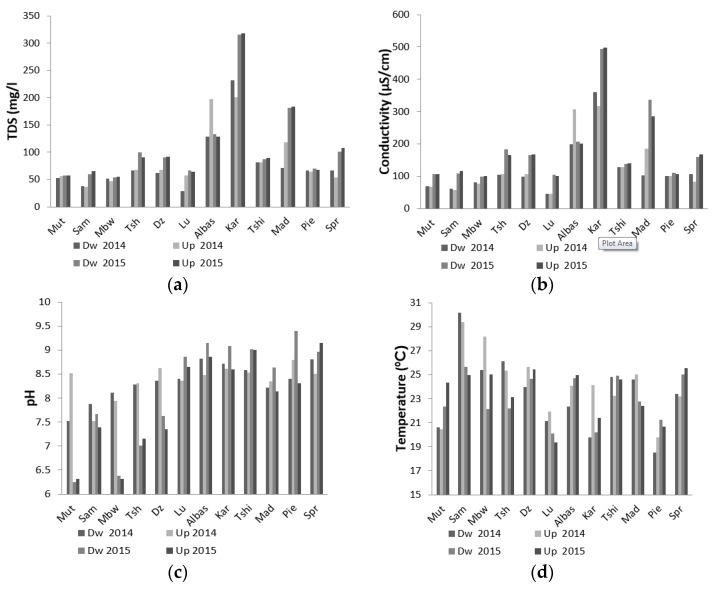
(**a**) Total Dissolved Solids (TDS mg/L); (**b**) Electrical conductivity (EC μS/cm); (**c**) pH; and (**d**) Temperature (Temp °C) determined on river water samples in 2014 and 2015.

The physical parameters determined on all water samples were within the acceptable limits recommended by the DWS and the WHO (Figure 2) [23,24]. Considering pH of 2014 samples (Figure 2c), a maximum value (8–8.5) was found for Up while for the Dw, the maximum values here 8.5–8.6. The pH variation was similar either upstream or downstream (*R*^2^ = 0.9072), showing stronger correlations closer to 1. A maximum temperature (30 °C, Figure 2d) was recorded in Sambandou water and a minimum (18–20 °C) in Luvuvhu. The maximum measurements for Conductivity (≥300 μS/cm, Figure 2b) were recorded in Albasini and Karringmelkspruit while conductivity measurement was low in Lutanandwa and Luvuvhu (Up). TDS was found to be >100 mg/L for all sites (Figure 2a). The variation in EC and TDS were similar either upstream or downstream (*R*^2^ = 0.9072), showing stronger correlations closer to 1.

In 2015, maximum pH measurements (≥8–9, Figure 2c) were recorded in Lutanandwa, Albasini, Karringmelkspruit, Madadzhe, Tshino, Piesanghoek and Springfield, while minimum pH reading (pH < 7) was recorded in Mutale and Mbwedi (both Dw and Up). A maximum temperature (25 °C, Figure 2d) was recorded in Sambandou (Dw) and for Dzindi and Tshino (Up) while minimum Temperatures measurements (18–19 °C) were recorded for Lutanandwa, Tshino and Karringmelkspruit (Dw) and Piesanghoek (Up). Conductivity values ≥400 μS/cm (Figure 2b) were recorded in Karringmelkspruit (Up) and Mutale, Mbwedi, Lutanandwa and Piesanghoek (Dw). Maximum values of TDS (>300 mg/L, Figure 2a) were recorded in Karringmelkspruit and Madadzhe (Up) whereas minimum TDS values (<100 mg/L) were seen in Mutale, Mbwedi, Lutanandwa and Piesanghoek (both Up and Dw).

### 3.2. Microbiological Results

TC MPN/100 mL at each sample point exceeded the recommended water quality standards. The concentration of TC was consistent between sampling points (Up and Dw) throughout the year with values above 2400 MPN/100 mL. The minimum TC values were recorded in Mbwedi, Lutanandwa and Albasini sites (Table 3).

Of all samples collected in 2014, 97.9% were found to be *E. coli* positive with 25% of the water samples with TC MPN/100 mL > 1000, 72.9% of sample with MPN/100 mL ranging from 10 to 1000 and only 2.1% of the water samples had counts within the range 0–10 MPN/100 mL. Madadzhe showed the highest contamination while Albasin Dam displayed the least *E. coli* contamination.

In 2015, TC reached maximum values of above 2400 MPN/100 mL for Karringmelkspruit, Luvuvhu and Madadzhe while a minimum was recorded in Albasini. *E. coli* counts greater than 2000 MPN/100 mL were observed in Madadzhe and Mutale while minimum values were observed in Sambandou, Tshinane, Lutanandwa, Albasisn and Tshino.

### 3.3. Pathogens Identified in Water Samples

The multiplex PCR was successfully run on all the samples and no PCR inhibition was observed (data not shown). All samples tested positive for the *mdh* gene confirming the presence of the *E. coli* microbiology results. A summary of the PCR results obtained is reported in Table 4 and shows that only 14 of all *E. coli* positive samples did not have pathogenic *E. coli*. The results further indicate that the targeted pathogenic *E. coli* strains could be detected in a variety of combinations (Table 4). This is not surprising since samples can theoretically contain possible mixtures of *E. coli* strains and the mPCR results rather describe the *E. coli* community enriched with the Colilert^®^ than the presence of a specific strain. Nonetheless, the results still indicate the presence of culturable pathogenic *E. coli* in the samples that might pose a risk to people using the water for bathing, food preparation and drinking if the water is used untreated.

### 3.4. Risk Activities in Rivers and Dams

Water samples were collected from 12 sampling sites and the risk activities that could affect water quality were recorded (Table 1). Eleven of the sampling sites, namely Mutale, Sambandou, Mbwedi, Tshinane, Dzindi, Lutanandwa, Karringmelkspruit, Luvuvhu-Tshino and Madadzhe, were flowing alongside residential areas, whereas Albasini Dam is situated in a non-residential area with large scale farming taking place. Activities observed in the 11 sampling sites situated in residential areas included car washing, animal grazing, drinking water collection, laundry, farming, bathing, baby diaper disposal, sewage discharge and the presence of fecal matter; the one outside the residential area showed farming activities and presence of fecal matter from domestic and wild animals.

The place where most of the activities (66.7%) were recorded was Mutale River. It was of concern to observe that Mutale river and Sambandou river were used by the community as a source of drinking water supply when one takes into account the high percentages of risk activities (Table 1). The presence of fecal matter from human, wild and domestic animals (91.7%) were amongst the top activities, whilst farming (58.3%) was the second highest activity. In this study, no chemical assessment was done to indicate its impact on water quality. However, the usage of detergents (soaps, washing powder) in certain activities such as car wash, body wash and laundry represented 33% risk for each activity because of the chemicals released into the water [25].

### 3.5. Risk Assessment and Seasonal Variation of Microbiological Water Quality Monitoring

To assess microbiological risks in the rivers and dam, the WHO risk assessment criterion was used indicating low risk contamination (1–3) when content equals 1 to 10 MPN/100 mL; intermediate to high risk (4–6) equates to 11 to 100 MPN/100 mL; and when MPN/100 mL is >100, the contamination risk is very high (7–10). In the dry and wet season, all the rivers and the dam were detected as having a very high risk (>100 MPN/100 mL) score (data not shown). In addition, in the wet season, TC and *E. coli* were present (>100 MPN/100 mL) in all sampling sites except the Albasini Dam. Samples from the Sambandou river and the Karingmelkspruit sampling point were classified as being very high risk category (>100 MPN/100 mL) when *E. coli* was monitored. Albasini Dam was the only site with low risks when compared to other sampling points in this study.

The elevated risks detected for *E. coli* (>100 MPN/100 mL) in the dry season were observed for most sampling sites except Tshinane, Lutanandwa, Luvuvhu-Tshino, Luvuvhu-Springfield and Albasin Dam. The cleanest site with low risk for *E. coli* (1–10 MPN/100 mL) was found to be Albasini Dam. Sambandou and Mutale Rivers, which were used as drinking water sources, scored very high risk.

### 3.6. Discussion

Temperature, electrical conductivity (EC), total dissolved solids (TDS) and pH may be critical for the growth and survival of bacteria in water. The pH range accepted for drinking water and domestic purpose by WHO is 6.5–8.5 [26]. In all the rivers surveyed, water pH ranged from 6.1 to 9.4 which are slightly below and above the accepted limit respectively. Based on these guidelines, the pH of the river water would not adversely affect its use for domestic and recreational purposes [27]. Catchments passing through farmlands (Karringmelkspruit, Springfield, Albasin, Piesanghoek) have pH levels above 8, and those passing through human settlements had pH values around 7. Based on this, it is evident that the pH of these sampling points is influenced by the nature of the deposits where the rivers pass through.

The temperatures of the river water ranged from 15 °C in winter to 30 °C in summer. These values were within the expected ranges during the sampling period. EC for all rivers was not affected by season. However, electrical conductivity in some rivers was significantly higher in winter than it was in summer. This difference could be attributed to accumulation of pollutants (as lower base flows in winter) that are washed away by rain. Generally, the conductivity of a river is lowest at the source of its catchments and, as it flows along the course of the river, it leaches ions from the soils and also picks up organic material from biota and its detritus [28]. The average value of unpolluted river water is approximately 350 μs/cm [29]. The Karringmelkspruit sampling point had the highest value, 488 μs/cm before rain and 502 μs/cm after rain, which indicated pollution in this river.

The fluctuations in EC correlated positively (not shown) with TDS [27]. In Karringmelkspruit water TDS content was higher than 300 mg/L in all seasons. Although the value is still below the WHO guidelines (1000 mg/L), there is still evidence of water pollution when looking at the percentage of risk activities [26].

Using the Colilert Quanti-Tray method to assess the concentrations (MPN/100 mL) of *Escherichia coli* (*E. coli* ) and total coliforms (TC) in the different river sampling points, the results confirmed the unsafety of these waters for drinking, as the estimated values do not fit the standards dictated by both DWAF and WHO [28,29]. This corroborates with previous studies in this region [30]. The levels of *E. coli* seemed to be influenced by the season in which the sampling was done. In all the samples collected there was a marked increase in the concentration of *E. coli* in the rainy season as compared with the dry season. The concentration of TC remained consistently high throughout the study period. TC represents a large group of bacteria of fecal origin (e.g., *Salmonella* spp., *Shigella* spp., *Vibrio cholera*, *Campylobacter*, etc.) [31]. These microbes are well known causes of diseases such as salmonellosis, dysentery, gastroenterititis, typhoid fever and cholera. Their presence in water in large numbers indicates an extremely high risk of transmission of infectious diseases.

Of the water samples collected, 97% were positive for *E. coli.* The remaining 3% had no *E. coli* and thus these waters were within the recommended water standards. A comparison of the concentrations of *E. coli* shows a general pattern in which samples collected from the downstream sites of the river have higher concentrations than those upstream. This is possibly due to an increase in human activity observed downstream. As seen during this study, most people used downstream water for washing clothes, bathing and as watering holes for the cattle since the river flow will have slowed down and the river could be easily be accessed for water collection. This might also be a contributing factor for the increase in *E. coli* counts at these sites.

The PCR results revealed the heavy contamination of water samples with all the pathogenic *E. coli* strains. The strains with high prevalence for both years were EAEC (87%), atypical EPEC (86%) and ETEC (83%). The results are extremely alarming considering how much of the contaminated water is used every day by villagers in the Vhembe district. The risk of transmission of enteric disease is therefore very high in this region.

The negative impact of contaminated drinking water on human health has been documented in many studies [3,4,32,33]. Human and animal activities were observed to play a role in the contribution of *E. coli* to the environment including in river waters [34].

Human activities taking place in the 12 sampling sites determined the contamination capacity and risk level. Mutale River (used as a source for drinking water) where activities such as sewage discharge, car washing, body washing, animal grazing and farming could be found, was the most contaminated river with very low pH and high TC and *E. coli* contents. The river is found to be the most contaminated sampling point. Most of the rivers which flowed within villages were found to be highly contaminated compare to rivers with minimal activities. These findings were similar to those of the study conducted in Kampala where the risk factors of microbial contamination of wells were determined [35]. In addition, most researchers have associated farming, the presence of animals next to rivers and sewage discharge with pathogen load [36,37,38].

The Vhembe district is primarily comprised of subsistence farmers who use the surrounding savannah for cattle grazing. The cattle, using the rivers as watering sources, normally produce dung (fecal matter) at the banks of these rivers resulting in contamination of the sources. It was also observed that poor hygiene practices could be sources of *E. coli* contamination of rivers. Dumping of disposable napkins and other garbage in the river sources also contribute not only to *E. coli* contamination but also to other pathogenic bacteria that can have a negative effect on human health [7].

It was of concern that rivers like Mutale and Sambandou were used as drinking water sources by the communities. The correlation between pH, EC and TDS indicated an increase in contamination from upstream to downstream. The conductivity was the only one that did not show correlation in the wet season between upstream and downstream with a high peak observed for the upstream point. This could indicate that salination upstream was higher, as reported in a study conducted in the North West [39]. The absence of *E. coli* in Albasin Dam could be the result of elevated pH as *E. coli* is known to be unable to survive in high alkaline water as compared to other bacteria.

It was noted that TC had a very high score (7–10) in both seasons, whereas *E. coli* content was high in six sampling sites in the wet season, whiles contamination was less in the dry season. Consequently, the contamination of the rivers was similar in both seasons; however, *E. coli* contamination was found to be moderate in the dry season. The presence of pathogenic *E. coli* in water is reportedly associated with diarrhea, usually linked to outbreaks in communities. If surface waters used by the communities for their domestic chores are continuously being misused without appropriate measures, it could result in major health problems.

## 4. Conclusions

The study showed that several of the rivers in the Vhembe District were polluted and were not fit to be used for human consumption. Various human activities usually take place at these sources which increase the contamination of water and seriously impact the quality of water. Many of these activities have been a way of life for people in rural areas for long periods of time. In a social context, people must be educated on risks and platforms for dialogue should be created. In addition, government authorities should step in and provide alternative solutions as well as assist the communities to take ownership of their drinking water resources. This study did not set out to look for solutions; instead, the aim was to see how certain activities could impact on the quality of the rivers. The results are clearly indicated in Table 1 as the percentage risk that these activities have on each site and also showed that fecal matter had 91.7% cumulative risk on all of these sites. The fact that diarrheal diseases are still in the top three leading causes of mortality and health issues in South Africa should be a warning sign for health authorities. More emphases need to be put on water contamination and proper hygiene practices.

## Figures and Tables

**Table 1:** Human activities observed at sampling sites.

Observed Risk Activities										
Sampling Sites	Car Wash	Animal Grazing	Drinking Water	Laundry	Farming	Body Washing	Sewage Discharge	Fecal Matter	Baby Diaper Washing	% of Risks Activities per Site
Mutale	X	X	X	-	X	X	X	-	-	66.7
Sambandou	-	X	X	X	-	-	-	X	-	44.4
Mbwedi	X	-	-	X	-	X	-	X	-	44.4
Tshinane	-	-	-	X	-	X	-	X	X	44.4
Dzindi	X	-	-	-	X	-	-	X	X	44.4
Lutanandwa	-	-	-	-	-	-	-	X	X	22.2
Albasin Dam	-	-	-	-	X	-	-	X	-	22.2
Karringmelkspruit	-	-	-	-	X	-	-	X	X	33.3
Tshino	X	-	-	X	-	X	-	X	-	44.4
Madadzhe	-	-	-	-	X	-	X	X	-	33.3
Piesanghoek	-	-	-	-	X	-	-	X	-	22.2
Springfield	-	-	-	-	X	-	-	X	-	22.2
Cumulative percent per risk activity	33.3	16.7	16.7	33.3	58.3	33.3	16.7	91.7	33.3	

- = No activity observed; X = Activity found.

**Table 2:** Bacterial strains used in molecular characterization [15].

Bacterial Strain	Reference	Use	Genes Present
*Escherichia coli* (Commensal) ^a^		PCR	*mdh*
Enterohaemorrhagic (EHEC)	ESCCO 21 ^b^	PCR	*mdh*, *stx1*, *stx2* and *eaeA*
Enteroinvasive (EIEC)	ESCCOS ATCC 43893 ^b^	PCR	*mdh* and *ial*
Enterotoxigenic (ETEC)	ESCCO 22 ^b^	PCR	*mdh*, *lt* and *st*
Enteropathogenic (EPEC)	S-ESCCO 16 Pl ^b^	PCR	*mdh*, *eaeA*, *bfp*
Enteroaggregative (EAEC)	ESCCO 14 ^b^	PCR	*mdh* and *eagg*
*E. coli*	ATCC 25922	Microbiology	
*Pseudomonas aeruginosa*	ATCC 31488	Microbiology	
*Klebsiella pneumoniae*	ATCC 10145	Microbiology	

^a^ Environmental isolate confirmed by API 20E (OMNIMED^®^, Moorestone, NJ, USA) and PCR as commensal *E. coli*; ^b^ Strains purchased from National Health Laboratory Services (NHLS) confirmed with biochemical and PCR by the NHLS.

**Table 3:** Total coliform (TC) and *E. coli* quantification of sampling points for 2014 and 2015.

Sampling Sites	Total Coliforms				*Escherichia coli*			
	Up 2014	Dw 2014	Up 2015	Dw 2015	Up 2014	Dw 2014	Up 2015	Dw 2015
**Mut**	2420	2420	2420	2420	163	643	1203.3	345
**Samb**	2420	2420	2420	2420	528	241	66	355
**Mbwe**	2420	1716	2420	2420	863	609	203	228
**Tshin**	2420	2420	2203	1595	241	247	114	85
**Dzin**	2420	2420	2203	2420	540	1517	214	242
**Lutan**	2420	1733	1917	2420	805	942	35	30
**Albas**	1812	1770	721	1700	40	12	6	6
**Karr**	2420	2420	2420	2420	227	141	157	206
**Tshino**	2420	2420	2420	2076	704	1250	275	178
**Mada**	2420	2420	2420	2420	2420	2420	2420	2420
**Pies**	2420	2420	1553	1378	212	143	125	126
**Spring**	2420	2420	1553	1393	331	372	21	19

Up = Upstream, Dw = Downstream.

**Table 4:** Relative percentages (%) of *E. coli* strains identified by PCR analyses.

Year	Com	aEPEC	tEPEC	EAEC	EHEC	EIEC	ETEC
2014	11 (22%)	14 (26%)	9 (18%)	17 (35%)	13 (27%)	3 (6%)	21 (43%)
2015	3 (6%)	31 (60%)	8 (15%)	27 (52%)	14 (27%)	8 (15%)	21 (40%)

Com = commensal, aEPEC = atypical Enteropathogenic *E. coli*, tEPEC = typical Enteropathogenic *E. coli*, EAEC = Enteroaggregative *E. coli*, EHEC = Enterohaemorrhagic *E. coli*, EIEC = Enteroinvasive *E. coli*, ETEC = Enterotoxigenic *E. coli.*
